# Immunohistological detection of pentosidine in the epiglottis:A contribution to molecular age estimation

**DOI:** 10.1007/s00414-026-03764-7

**Published:** 2026-03-18

**Authors:** Sandra Schneider, Rim Ouannane, Lisa König, Stefanie Ritz

**Affiliations:** Institute of Legal Medicine, University clinic Düsseldorf, Düsseldorf, Germany

**Keywords:** Age estimation, Advanced Glycation End Products (AGEs), Pentosidine, Epiglottis, Immunohistochemistry

## Abstract

Pentosidin (Pen) accumulates in various tissues with increasing age, among others in the epiglottis. So far, all published Pen concentrations in the epiglottis are based on the analysis of total tissue samples via HPLC. The morphological structures and proteins in which Pen accumulates remain unknown. Such insights would be of great importance for optimizing Pen based methods for age estimation, especially to overcome the problem of sample inhomogeneity in older individuals as source of error in age estimation based on Pen.

Against this background, pentosidine was detected immunohistochemically in epiglottis tissue samples from 54 deceased individuals (age range: 6.5 months to 90 years). Staining intensity and intratissue distribution were evaluated.

Pen was localized almost exclusively in the elastic cartilage of the epiglottis. Positive staining was most pronounced in fibers surrounding the chondrocytes, the staining pattern suggested a three-dimensional fiber network with a particularly dense fiber arrangement around these cells. Very similar findings were observed using Elastica-van Gieson staining of elastic fibers.

Pen could not be detected immunohistologically in samples of young individuals up to an age of c. 35 years. From the mid-30s onward, staining became more frequent, with both staining intensity as well as the expansion of the areas with positive findings increasing with age.

The observed immunohistological staining pattern provides new insights into Pen accumulation in the epiglottis. It is consistent with the assumption that Pen accumulates almost exclusively in elastic fibers, and here with a very high probability in elastin. The accuracy of age estimation based on Pen could be significantly improved if not - as is currently the case - total tissue samples are examined, but rather purified proteins in which Pen accumulates; elastin in the epiglottis was identified as a target protein for such an approach.

## Introduction

Molecular methods are increasingly becoming part of the methodological repertoire of postmortem age estimation. The most promising molecular methods are based on DNA-methylation [1–5] and post-translational protein modifications, such as the accumulation of D-aspartic acid and pentosidine [6–8]. These molecular methods promise more accurate results, particularly in adults, than conventional methods which are mainly based on the examination of physiological and degenerative changes (especially in dental and skeletal structures) associated with aging. The applicability of the various methods for age prediction can be influenced and limited by many factors, for example, advanced postmortem changes (e.g. putrefaction, skeletonization), exposure to heat, and the lack of tissue availability in isolated body parts. The selection of the best method must be based on the specific circumstances of each individual case.

Pentosidine (Pen) is an advanced glycation end product (AGE). AGEs accumulate in long-living proteins during lifetime under physiological conditions as well as under specific pathological conditions as long-lasting hyperglycemic states or renal failure [9–12]. Most data on the accumulation of AGEs in human tissues originate from basic research on the pathogenesis of typical age-related diseases and in particular the late complications of diabetes mellitus. The accumulation of AGEs may cause various disorders in tissues and cells and contributes to the pathology e.g. of diabetes mellitus, atherosclerosis and osteoporosis [13–23].

Pentosidine accumulates during life time as a fluorescent crosslink between arginine and lysine in many proteins, e.g. in collagen [24, 25], elastin [26, 27], lens crystallins [28, 29], proteoglycans (e.g. aggrecan) [30] or basement membrane proteins (e.g. laminin, fibronectin) [31–33]. An age dependent accumulation of Pen was observed in dentine [34, 35], achilles tendon [36, 37], intervertebral disc [38, 39], bones (bone samples from skull, rib, and clavicle) [6, 7, 34], epiglottis [38], eye lens [28, 29, 40] and skin [24, 41, 42].

Meanwhile, several studies have investigated the potential applicability of Pen accumulation in human tissues for forensic age estimation [6, 8, 34, 35, 43]. The analysis of Pen can be used for age estimation in cases in which confounding factors as long-lasting hyperglycemic states or renal failure can be excluded or as an additional parameter, e.g. in combination with D-aspartic acid [7, 38, 44, 45]. It has been shown that such a combined use of parameters in multivariate models can optimize age estimation [1, 7, 35, 38]. A recent systematic review supports this approach [45]: While age estimation based on DNA methylation currently offers the highest individual accuracy under optimal conditions (e.g. short postmortem interval, no advanced putrefaction), multivariate approaches – such as the combination of pentosidine, D-aspartic acid and methylation data – show the greatest potential under forensic casework conditions [45]. These approaches capture complex aging processes more precisely, better compensate for confounding antemortem or postmortem factors, and thus significantly increase the accuracy of forensic age determination [22]. In such multivariate approaches, the inclusion of biological information from Pen data in models is useful. Of further practical relevance is the finding that Pen – in contrast to DNA methylation and D-aspartic acid – appears to be stable for a very long time postmortem, possibly for thousands of years [1, 8].

However, the use of Pen as a single parameter for age estimation is currently limited by a considerable scatter of values in most tissues, at least in older individuals, which may not be (solely) due to the influence of confounding factors [38, 41, 44, 46]. Rather, the composition of the samples examined may also play a role. So far, only total tissue samples (and not purified proteins) have been examined to evaluate the usability of Pen for forensic age estimation. It can be assumed that each protein has its own kinetics of Pen accumulation. Given the known increasing instability of tissue homeostasis with age, the higher scatter of Pen values ​​observed in older adults could be largely due to the increasing inhomogeneity in the tissue samples with age [47–50]. The analysis of purified proteins with age-dependent accumulation of Pen could solve this problem. However, this would require the identification of suitable target proteins.

A first step toward identifying such target proteins can be the immunohistological localization of Pen in tissue samples – this was the aim of the study. Therefore, immunohistological studies with an anti-Pen antibody were conducted on epiglottis tissue, which exhibits an age-dependent accumulation of Pen with considerable variation in older age, when examined as whole tissue via HPLC [44].

## Materials and methods

Pentosidine was detected immunohistochemically in epiglottis tissue sections collected during autopsy in the Institute of Forensic Medicine at the University Hospital Düsseldorf. The project was approved by the Ethics Committee of the Medical Faculty of Heinrich Heine University Düsseldorf.

### Tissue samples

A total of 54 epiglottis tissue samples were collected from 16 females and 38 males in the age between 6.5 months and 90 years. Individuals with known diabetes mellitus disease and chronic renal failure were excluded. Tissue samples were obtained from the base of the epiglottis. Samples with pronounced postmortem tissue changes were excluded. The collected samples were fixed in formalin and embedded in paraffin.

### Immunohistochemistry (Anti-Pen)

Tissue sections were deparaffinised and rinsed in distilled water. Antigen retrieval was carried out with proteinase K for ten minutes. The sections were rinsed twice for five minutes with TBS buffer. They were blocked for ten minutes in a humid chamber using a peroxidase inhibitor. The sections were rinsed again twice with TBS buffer for five minutes. The primary antibody against pentosidine (Anti-Pentosidine Monoclonal Antibody Clone No. PEN-12, Trans Genic Inc.) was used at a concentration of 1:200. The sections were then incubated with this primary antibody for two hours in the Autostainer i6000. This was followed by incubation with the secondary antibody (Histofine M) for one hour. The sections were stained in DAB for seven minutes. After staining, they were rinsed in distilled water for two minutes. The sections were stained with hemalum for 30 s, then rinsed with distilled water. For differentiation, they were rinsed in HCl and rinsed again in distilled water for five minutes. Finally, the sections were coverslipped with Aquatex.

Tissue from an 87-year-old individual with a high Pen content in the HPLC analysis served as a positive control and was stained in each batch.

### Elastica-van Gieson (EvG) staining

Elastica-van Gieson (EvG) staining was used to visualize elastic fibers. After deparaffinization and rehydration, the sections were treated with Weigert’s resorcinol-fuchsin solution (staining time: 15 min), which stained the elastic fibers dark purple to black. Subsequently, counterstaining with van Gieson’s solution was performed, which contrasted collagen fibers red and cytoplasm yellow. EvG staining allowed for clear delineation of the elastic networks, particularly in the pericellular area and along the connective tissue septa of the epiglottic cartilage. The resulting histological orientation was used to assign the immunohistochemical pentosidine signals to the anatomy and matrix.

All histological stainings were performed on consecutive sections of the identical specimens to allow a spatial correlation between the immunohistochemically detected pentosidine and corresponding tissue structures.

### Image acquisition and evaluation

Microscopic documentation was performed using a Nikon Eclipse Ci light microscope, and photographs were taken using the NIS-Elements D5.02.00 imaging software.

Staining intensity, intratissue distribution and association of the immunohistologically stained structures with the findings in the EvG staining were evaluated.

The staining signals were described semiquantitatively using a 3-point score (0 = negative, 1 = weak to moderate, 2 = strongly positive).

All sections were independently evaluated by two investigators. In cases of ambiguity or in cases of disagreement, a third investigator was involved to achieve consensus.

## Results

### Pen accumulates in the elastic cartilage in the epiglottis

**Fig. 1:**
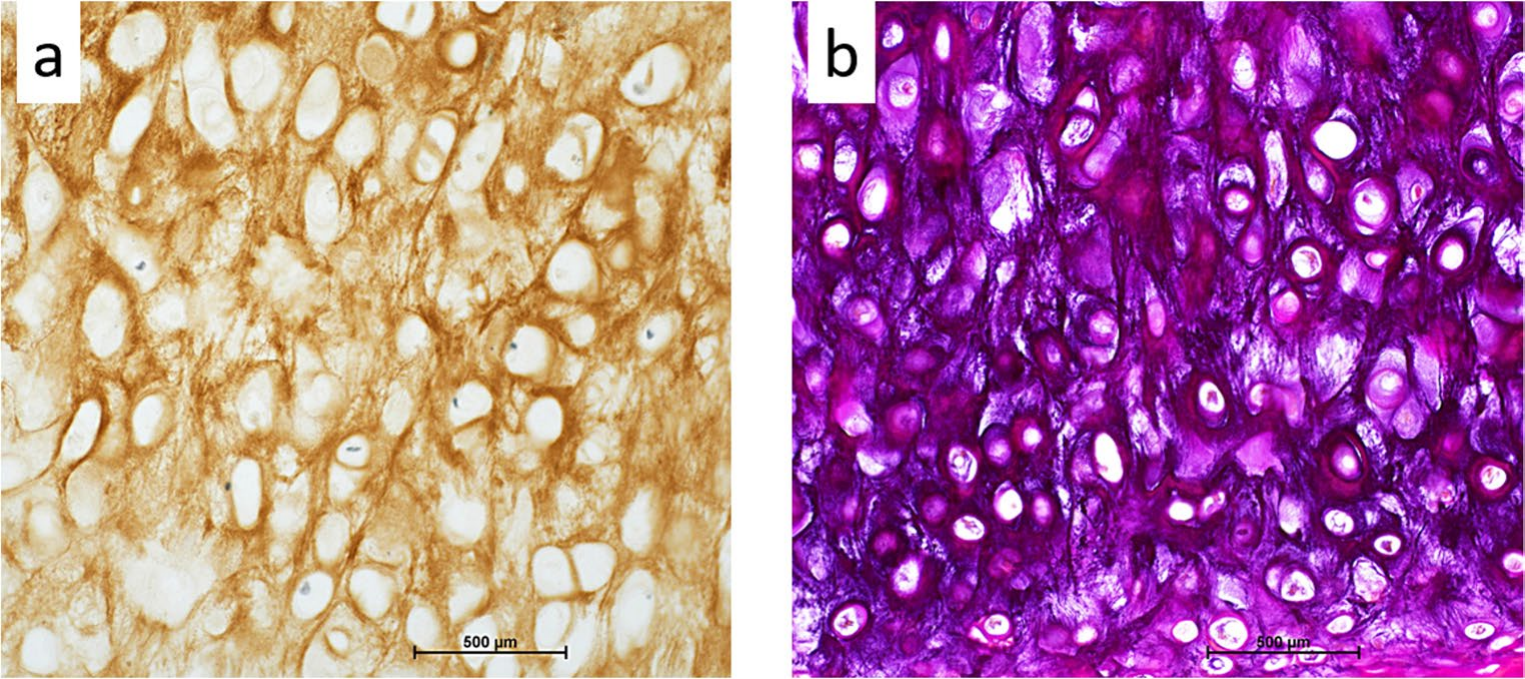
Immunohistochemical localization of pentosidine and histological visualization of elastic fibers in the epiglottis: (**a**) Immunohistochemical staining with Pentosidine Monoclonal Antibody Clone No. PEN-12, Trans Genic Inc demonstrates pentosidine accumulation in the elastic cartilage of the epiglottis, predominantly in fibers surrounding the chondrocytes (200× magnification). (**b**) Corresponding section stained with Elastica-van Gieson (EvG) highlights the elastic fiber network (200× magnification)

Pen was localized almost exclusively in the elastic cartilage of the epiglottis **(**Fig. 1**)**. Interstitial tissues (such as loose connective tissue and mucosa) and chondrocytes showed no staining. Occasional isolated staining was observed in the mucosal epithelium.

### Pen accumulates mainly in fibers surrounding the chondrocytes

Positive staining was most pronounced in fibers surrounding the chondrocytes, the staining pattern suggested a three-dimensional fiber network with a particularly dense fiber arrangement around the chondrocytes **(**Fig. 1**)**.

### Pentosidine accumulates in elastic fibers

The localization of pentosidine showed a strong overlap with the distribution of elastic fibers as confirmed by Elastica-van Gieson staining **(**Fig. 1**)**.

### The intensity of staining as well as the stained areas increased with age

**Fig. 2:**
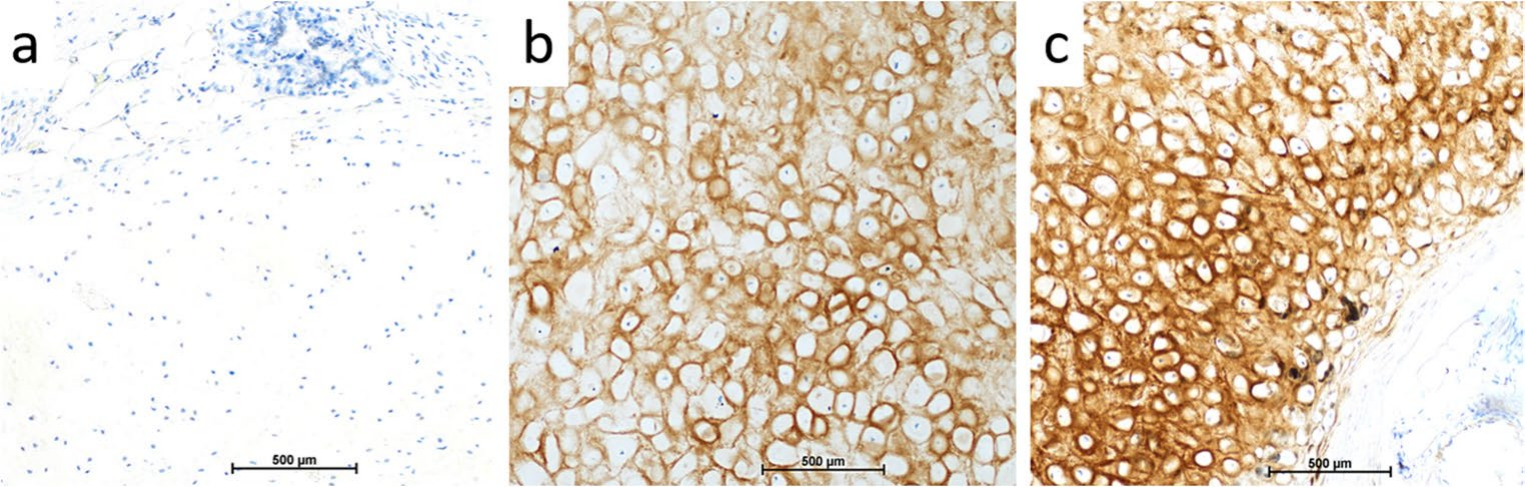
Typical immunohistological staining of epiglottis samples from individuals of different ages with Pentosidine Monoclonal Antibody Clone No. PEN-12, Trans Genic Inc (100x magnification): (**a**) Sample of a 19-year-old individual showing no detectable pentosidine; (**b**) sample of a 43-year-old individual displaying moderate, island-like staining; (**c**) sample of an 81-year-old individual showing strong staining

**Fig. 3:**
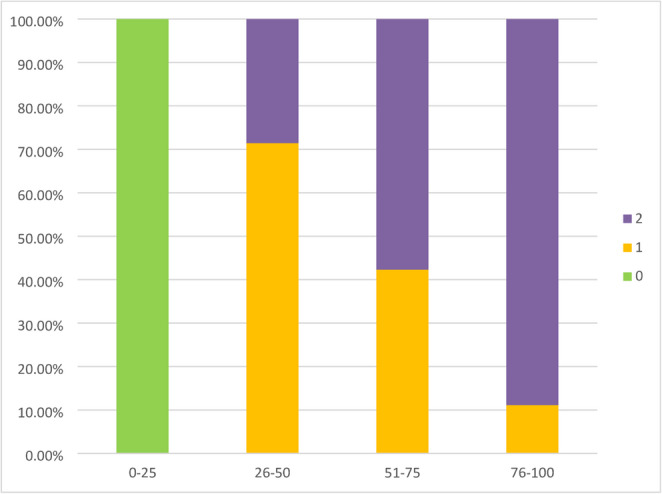
Staining intensity in predefined age groups. The x-axis represents age ranges in years (0–25, 26–50, 51–75, 76–100), the y-axis indicates the percentage of samples assigned to each category of a three-point staining intensity scoring system. Score 0 corresponds to no staining, score 1 to low to moderate staining, and score 2 to strong staining

Pen could not be detected in samples of children, adolescents and young adults up to an age of c. 35 years. However, no samples were available for individuals aged 26–35 years. From the mid-30s onward, staining became more frequent. In mid-aged individuals, staining often appears in an island-like pattern, in higher ages it became more and more extensive and rich in contrast. Overall, the staining intensity showed an age-dependent increase. Low to moderate staining (score 1) was most frequently observed from the mid-30s to the early 50s. In an individual case, strongly positive staining (score 2) was already present in younger age (43 years old). In the age range between mid-50s and early 70s, considerable interindividual variability was observed, with strongly positive staining of score 2 predominating in individuals older than 75 years. From the age of 80, strongly positive staining of score 2 occurred almost exclusively **(**Figs. 2a–c and 3**)**. Within the limitations imposed by the sample size and the unequal distribution between sexes, we did not observe any significant differences between male and female cases.

## Discussion

Previous work on Pen concentration in the epiglottis (quantified by HPLC) has already shown, that Pen accumulates in this tissue with age. This age dependent accumulation of Pen could be demonstrated also immunohistologically; the findings allow for a rough categorization in age groups: No positive detection of Pen – age most likely under 35 years, positive Pen detection with low to moderate contrast staining – age most likely between the mid-30s to the early 50s, positive Pen detection with strongly positive contrast staining – age likely over 75 years, more likely older than 80 years **(**Figs. 2a–c and 3**)**. The focus of this study was not on establishing an immunohistological method for age estimation. It remains to be clarified, if more precise immunohistological age diagnoses can be achieved by optimizing staining and analysis of staining results (e.g., with automated, AI-supported systems). However, quantification by HPLC will certainly remain the gold standard for age determination based on Pen.

The main aim of this work was to localize Pen in the epiglottis tissue – as a first step to overcome the problem of sample inhomogeneity as source of error in age estimation based on Pen. Since this problem can only be solved by analyzing purified samples, a suitable target protein should be identified. According to our immunohistological results, Pen accumulates in the elastic fibers of the elastic cartilage in the epiglottis. The main protein in the elastic fiber is elastin. Elastin is considered one of the longest-lived proteins in the human body. Based on the age-dependent accumulation of D-aspartic acid in yellow ligament specimens, in the skin, and in the arterial wall [51–53], elastin has already been identified as an age-dependent, long-lasting protein. Therefore, it seems justified to identify elastin as a target protein to be purified for age estimation based on Pen.

Our findings are consistent with previous studies describing a Pen accumulation in long-lasting matrix proteins such as collagen and elastin [26, 54]. From a basic scientific perspective, our findings can be interpreted as an indication of an increasing degeneration and ”aging” of the elastic fibers. Pentosidine is known to form covalent crosslinks between lysyl and arginyl residues of neighboring protein chains, thereby decreasing the elasticity of the matrix. An increased crosslink density could thus lead to reduced extensibility and increased stiffness of the cartilage. In the context of the epiglottis, this could promote an age-dependent impairment of flexibility and protective function during swallowing. Similar AGE-associated changes have been described in hyaline cartilage, where pentosidine reduces mechanical load capacity through collagen cross-linking [55]. In other tissues, such as tendons [56] and blood vessels [57], increased AGE exposure also leads to stiffness, loss of elasticity, and functional limitations.

The identification of elastin as a long-lived protein with Pen accumulation could be an important step towards optimizing molecular age determination. If elastin can be purified to sufficient quality, the quantification of Pen (using HPLC) in purified elastin could enable significantly more precise age determinations than the current analysis of whole tissue. The usefulness of such a strategy has already been demonstrated for age estimation based on the accumulation of D-aspartic acid: As compared to the analysis of total bone proteins, the analysis of the purified bone peptide osteocalcin can significantly improve the precision of age estimation, even in older individuals [58]. And after elastin purification, a strong correlation between D-aspartic acid content and chronological age was also observed in skin, arterial walls, and yellow ligaments [51–53]. Elastin will be our target protein for a further optimization of age estimation based on Pen in the epiglottis.

## Conclusion

The observed immunohistological staining pattern provides new insights into the age dependent accumulation of Pen in the epiglottis. Pen accumulates in elastic fibers; notably, elastin is long-living protein that exhibits an accumulation of Pen during lifetime. The analysis of Pen in purified elastin (instead of total tissue samples) could significantly improve age estimation based on Pen in the epiglottis. This hypothesis should be further tested in subsequent studies.

## Data Availability

The datasets generated during and/or analysed during the current study are available from the corresponding author on reasonable request.

## Declarations

In partial fulfillment of the requirements for a doctoral degree.
